# Skin Prick Test Analysis in Allergic Rhinitis Patients: A Preliminary Study in Abuja, Nigeria

**DOI:** 10.1155/2016/3219104

**Published:** 2016-05-10

**Authors:** P. U. Ibekwe, T. S. Ibekwe

**Affiliations:** University of Abuja Teaching Hospital, PMB 228, Garki, Abuja, Nigeria

## Abstract

Allergic rhinitis (AR) is prevalent in Nigeria, though little information exists on the allergen. We assessed the clinical features of AR patients in our environment based on the allergic rhinitis impact on asthma (ARIA) classification. Only patients with positive skin prick test (SPT) were recruited. Seventy-four patients participated in the study. AR and asthma comorbidity were observed in 13.5%. The proportion of “sneezers-runners” was higher than “blockers” with significantly more “sneezers-runners” having persistent AR (*P* = 0.007). No relationship was established between these predominant symptoms and the aeroallergens used in this study. Intermittent mild and moderate/severe AR were evident in 13.5% and 31.1%, while persistent mild and moderate/severe were seen in 20.3% and 35.1%, respectively. House dust mites allergen yielded the highest number of positive responses (22.6%) followed by tree pollen (16.8%). No relationship was observed between the allergens tested and AR severity. Majority of patients were oligosensitive (33.8%) and polysensitive (35.1%) and were not significantly associated with AR severity (*P* = 0.07). Most AR patients presenting for treatment in Abuja, Nigeria, had moderate-severe persistent AR and showed similar SPT sensitization pattern with countries having similar climatic conditions. Sensitization patterns were not related to ARIA classification or predominant AR symptoms.

## 1. Introduction

Allergic rhinitis (AR) is a recurrent or chronic allergen specific, IgE-mediated hypersensitivity disorder affecting the nasal lining and characterized by nasal congestion, rhinorrhea, sneezing, nasal itchiness, and/or postnasal drip [1]. It is common worldwide and significantly impairs the quality of life of affected persons [2]. However, it is still underdiagnosed and undertreated in many countries [3]. In Nigeria, limited studies on AR exist and epidemiological studies based on the allergic rhinitis and its impact on asthma (ARIA) criteria are lacking. According to ARIA guidelines, allergic rhinitis is defined if two or more symptoms of rhinorrhea, nasal itching, nasal blockage, or sneezing are present in a patient for at least one hour per day for 4 days or more a week and also for 4 or more weeks a year [4]. Based on duration, symptoms are intermittent (<4 days/week or <4 weeks/year) or persistent (>4 days/week or >4 weeks/year). Severity grading is either mild or moderate-severe based on the absence or presence of sleep disturbance and impairment in daily activities, school, and work, respectively [1].

Skin prick test (SPT) is a standardized test widely used in the diagnosis of suspected cases of IgE-mediated allergy. It is considered as the gold standard in the diagnosis of allergy [3]. Generally accepted indications for SPT include allergic rhinitis, asthma, atopic dermatitis, suspected food allergies, latex allergy, and conditions in which specific IgE is suggested to play a role in the pathogenesis. It provides information about the presence of specific IgE to protein and peptide antigens (allergens). Identification of common aeroallergens in an area is necessary, in order to educate the patient on what allergens to avoid and also help find the best formulation of allergen immunotherapy for effective AR treatment.

To date, there has been no information regarding the common allergen from the Federal Capital Territory (FCT) of Nigeria, Abuja. The aim of this study is to identify the clinical profile of AR patients according to ARIA guidelines and investigate the common allergens in Abuja, Nigeria.

## 2. Materials and Methods

This cross-sectional study was conducted for a period of 18 months (March 2014 to September 2015). Sample population was based on the AR patients referred to the allergy clinic, affiliated to Medicaid Radiodiagnostic Center, Wuse 2, Abuja. Patients were consecutively recruited (convenient sampling method) and fall within the age range of 5 to 65 years. They were patients with a positive history of nasal inflammation (at least 2 or more of the following symptoms: rhinorrhea, sneezing, nasal blockage, nasal itchiness, and postnasal drip) for at least one-year duration. Patients' symptoms were categorized as “sneezers-runners” and “blockers” based on the predominant complaint [5]. Patients whose chief complaints include sneezing, rhinorrhea, and itchy eyes and nose were classified as “sneezers-runners,” while those with nasal blockage, postnasal drip, and difficulty with breathing were classified as “blockers.”

SPT was performed on patients who have stopped taking antihistamines at least 5 days before the test, while patients with severe dermatographism were excluded from the study. Informed consent was obtained from all patients. SPT was performed by the same investigator.

A total of 22 allergens were used in this study; these allergens make up the subtropical prick test batch of ALK-Abello, Denmark. These include tree pollen (oak, pecan, black willow, pine, cypress, red cedar, and box elder), weed pollen (pigweed, ragweed, and plantain), grass pollen (bermuda, bahia, johnson grass, and grass mix (meadow, orchard, timothy, june, rye, and redtop)), house dust mites (*Dermatophagoides pteronyssinus* and* Dermatophagoides farinae* mix), molds (*Alternaria tenuis*,* C. cladosporioides*,* Penicillium* mixed, and* Aspergillus* mixed), animal dander (cat hair and dog epithelium), and cockroach extracts. Of the pollen allergen used in this study, about 80% of the plants are present in Abuja environment. SPT was performed according to international guidelines [6] as a one-time test done on two forearms with lancets and allergens (ALK-Abello skin prick test kit, Berge Alle, 2970 H*ø*rsholm., Denmark) placed at least 2 cm apart to avoid contamination. A positive reaction is a wheal ≥ 3 mm in diameter. Histamine hydrochloride (1%) and normal saline (0.9%) were used as positive and negative controls, respectively. Patients with negative skin prick test were excluded from the study.

The patients' data was classified according to the ARIA guidelines and SPT results were analysed in allergen-clusters of tree pollen, weed pollen, grass pollen, house dust mites (HDM), molds, animal dander, and cockroach extracts. SPSS 16 software (Chicago Illinois) was used in the analysis and *P* value of less than 0.05 was considered significant.

## 3. Results

A total of 96 new patients with suspected allergic rhinitis presented at the allergic clinic within the study period. Only 74 patients from these had a positive SPT result and were enrolled into the study. There were more females (56.8%) than males. The youngest was 5 years and the oldest was 65 years. Their mean age was 30.8 years (95% CI 26.7 to 34.9 years). Twenty-one respondents (28.4%) were categorized as children (5 to 17 years of age) with a male : female ratio of 1.3 : 1. The majority of the study population resided in Abuja Municipal Area Council (AMAC) territory which is basically the city center.

Table 1 summarizes the main clinical features of the patients. The prevalence of asthma, urticaria, and conjunctivitis as comorbidity was lower when compared with comorbidity such as hypertension. Asthma was comorbidity in 10 patients (13.5%). Seven patients had persistent nasal symptoms postadenoidectomy. Positive family history of atopy was seen in 56.8% of patients and 20.2% of the subjects had animal contact within their environment.

According to predominant symptoms, the proportion of “sneezers-runners” was higher than “blockers” (56.8% versus 43.2%). “Sneezers-runners” tend to have persistent AR, while “blockers” symptoms were more intermittent (*P* = 0.007). Based on ARIA guidelines, most patients (67.2%) had moderate-severe AR (intermittent and persistent) and this was significantly related to animal exposure (*P* = 0.035) and not to age, gender, or family history of atopy. There was no significant relationship between these predominant symptoms and any of the aeroallergens.

There was a significant association between AR severity and the predominant complaints (Table 2) by the patients (*P* = 0.005). Moderate-severe persistent AR was more common among “sneezers,” while moderate-severe intermittent AR was common with the “blockers.” There was no significant association between AR severity and the presence of asthma (*P* = 0.26) or family history of atopy (*P* = 0.19).

**Figure 1:**
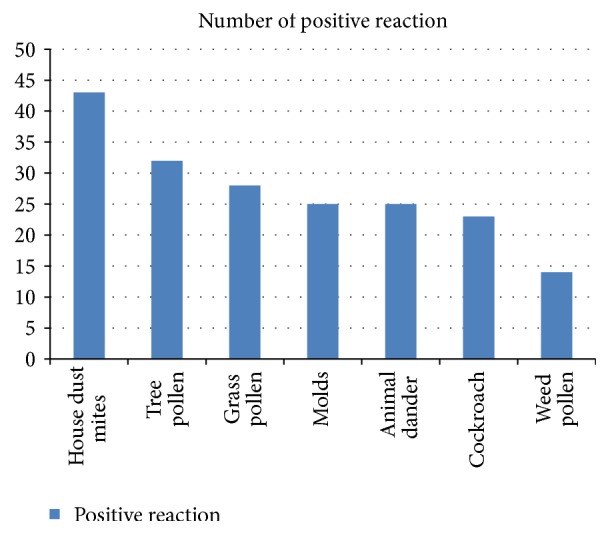
It illustrates the distribution of aeroallergens to which allergic rhinitis patients were sensitized to.

**Figure 2:**
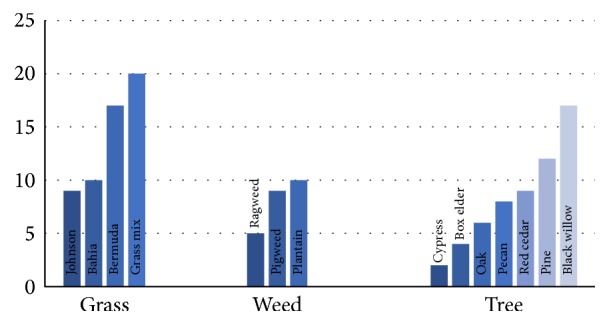
It shows the frequency distribution of individual pollens tested.

House dust mites allergen yielded the highest number of positive responses (22.6%) followed by tree pollen (16.8%). Weed pollen allergen yielded the least (7.4%), while animal dander and fungi allergen both came to 13.1% each (Figure 1). The sensitivity pattern of different pollens tested is shown in Figure 2. Mold sensitivity significantly affected more adults than children (*P* = 0.03). Also, house dust mites was significantly related to a positive family history of atopy (*P* = 0.035). There was no significant difference between the positive skin tests and gender as well as history of asthma. Only 23 of the 74 patients (31.1%) had sensitivity to one allergen. There was no single allergen that has the tendency for monosensitization.

Furthermore, no relationship was observed between the allergens tested and duration of AR (intermittent and persistent), as shown in Table 3.

Most of the patients with a positive SPT were in the persistent AR category (66.2%). The number of allergens which produced a positive skin response from each patient was closely distributed. The highest was reaction to 3 or more allergens (35.1%) followed by reaction to 2 allergens (33.8%) and then to one allergen (31.1%), as shown in Table 4. Statistical analysis did not reveal a relationship between AR severity and skin prick test reactivity.

## 4. Discussion

The prevalence of AR is increasing worldwide, yet it remains underdiagnosed and undertreated especially in developing countries [3]. A self-reported survey of AR among adult Nigerians observed a prevalence of 29.6% and a mean age of 29.3 years [7] which was close to the mean age observed in this study. The number of patients in this study with concomitant asthma is lower than other Nigerian studies [7, 8] and this could be due to small sample size or poor awareness of asthma symptoms by the respondents. Desalu et al. [7] observed a low level of awareness of asthma by patients in Nigeria, such that most people with asthma symptoms do not present to the physician but prefer unorthodox means of medical care.

About a third of the children (33%) in this study reported persistence or recurrence of rhinitis symptoms after adenoidectomy. This was lower than was observed by Colavita et al. [9] but significant enough to encourage more research with a larger sample size. The slight female predominance observed in our adult AR patients is in consonance with the findings in Malaysia and India [3, 5]. We also corroborated the study that showed a higher male predominance of AR among children [3].

We recorded a higher proportion of “sneezers-runners” to “blockers” similar to the findings by Lee et al. [10] and Shah and Pawankar [11] but different from study by Deb et al. [5]. This was significantly related to ARIA classification with more “sneezers” having persistent AR and more “blockers” having intermittent AR (*P* = 0.007). We could not establish any relationship between these predominant symptoms and the aeroallergens used in this study. Studies by Shah and Pawankar [11] and Deb et al. [5] observed that “blockers” were more sensitized to HDM, house dust, and fungi, while “sneezers” were more sensitized to pollens.

This study observed that a majority of AR patients were categorized as moderate-severe persistent AR, according to ARIA classification, while the least were mild intermittent AR. This has been observed by most studies [3, 5, 12] with warm climate like Nigeria. A constantly high environmental temperature and humidity could lead to a persistently high concentration of indoor and outdoor allergens all year round [3]. There could also be a selection bias, since patients would more likely present for treatment when their condition is severe and persistent.

No relationship was established between the type of AR and the allergen to which the patients were sensitized. This was similarly observed in a national, cross-sectional study of AR patients in Mexico [13]. The most common aeroallergen was house dust mites, followed by tree pollen as was seen in other SPT studies in Nigeria [8, 14–16]. However, there are no AR studies in relation to tree pollen sensitivity to compare with. This study highlights the importance of pollen allergens among AR patients living in Nigeria, a tropical country with high humidity. This supports an earlier observation of increased tree pollen sensitization in tropical environment [13] and emphasizes the need for increased research in this aspect. Further studies are needed to record the season of pollination of the different pollens found in Abuja and to correlate these findings with the timing of symptoms in sensitized patients.

Our study also revealed a tendency for multiple sensitizations for allergens among the patients. This was statistically significant in all except animal dander. Thus a patient with positive sensitization to house dust mites could also be sensitized against pollen, insect, or fungi allergens. This supports the argument that time of exposure (seasonal or perennial) does not properly define AR patients [10] and also creates difficulty with regard to immune therapy via hyposensitization. In addition, there is need for the use of SPT that incorporates wide variety of allergens within a specific environment in order to avoid skipping some of the sensitive allergens attributed to each individual. This will ensure a holistic treatment of the AR and better outcome.

In conclusion, most AR patients presenting for treatment in Abuja, Nigeria, have moderate-severe persistent AR and show similar SPT sensitization pattern with other countries having similar climatic conditions. Sensitization patterns are not related to ARIA classification or any predominant AR symptoms but rather may rely on the environmental condition of study area and genetic makeup of the study population.

## Figures and Tables

**Table 1:** Clinical characteristics of the patients (*n* = 74).

	Number (total = 74)	Percentage (%)
Gender		
Male	32	43.2
Female	42	56.8
ARIA classification		
Mild intermittent^a^	10	13.5
Moderate-severe intermittent	23	31.1
Mild persistent^b^	15	20.3
Moderate-severe persistent	26	35.1
Associated morbidity		
Urticaria	4	5.4
Asthma	10	13.5
Conjunctivitis	3	4.1
Posttonsillectomy	7	9.5
Sinusitis	3	4.1
Hypertension	12	16.2
Family history of atopy	42	56.8
Animal exposure	15	20.3
Carpet exposure	33	44.6
Major symptoms of patient		
Sneezers	42	56.8
Blockers	32	43.2

^a^Intermittent symptom <4 days/week or <4 consecutive weeks.

^b^Persistent symptoms >4 days/week or >4 consecutive weeks.

**Table 2:** Prevalence of clinical symptoms according to ARIA classification.

Symptom	Mild intermittent	Moderate-severe intermittent	Mild persistent	Moderate-severe persistent	Total
Blockers	7	11	6	8	32
Sneezers	3	4	17	18	42

Total	10	15	23	26	74

Intermittent symptom <4 days/week or <4 consecutive weeks.

Persistent symptoms >4 days/week or >4 consecutive weeks.

*χ* ^2^ 12.85, df 6, and *P* = 0.005.

**Table 3:** Relationship between allergens and duration of AR.

Allergen	Intermittent AR	Persistent AR	*P* value
House dust mites (*n* = 43)	12	31	0.05
Tree pollen (*n* = 48)	13	19	0.81
Weed pollen	5	9	1.00
Grass pollen	9	19	0.47
Fungi (*n* = 25)	6	19	0.13
Animal dander (*n* = 25)	11	14	0.46
Cockroach (*n* = 23)	8	15	0.79

**Table 4:** The distribution of skin prick test reactivity (based on allergen cluster) according to ARIA classification.

ARIA classification	One allergen (monosensitive)	2 allergens (oligosensitive)	3 or more allergens (polysensitive)	Total
Mild intermittent	4	6	0	10 (11.1%)
Moderate-severe intermittent	7	5	3	15 (16.7%)
Mild persistent	5	6	12	23 (31.1%)
Moderate-severe persistent	7	8	11	26 (35.1%)

Total	23 (31.1%)	25 (33.8%)	26 (35.1%)	74 (100%)

Intermittent symptom <4 days/week or <4 consecutive weeks.

Persistent symptoms >4 days/week or >4 consecutive weeks.

*χ* ^2^ 11.50, df 6, and *P* = 0.07.
